# Loss of retinal ganglion cells in a new genetic mouse model for primary open‐angle glaucoma

**DOI:** 10.1111/jcmm.14433

**Published:** 2019-05-29

**Authors:** Sabrina Reinehr, Dennis Koch, Maximilian Weiss, Franziska Froemel, Christina Voss, H. Burkhard Dick, Rudolf Fuchshofer, Stephanie C. Joachim

**Affiliations:** ^1^ Experimental Eye Research Institute University Eye Hospital, Ruhr‐University Bochum Bochum Germany; ^2^ Institute of Human Anatomy and Embryology University Regensburg Regensburg Germany

**Keywords:** electroretinogram, primary open‐angle glaucoma, retinal ganglion cells, βB1‐CTGF

## Abstract

Primary open‐angle glaucoma (POAG) is one of the most common causes for blindness worldwide. Although an elevated intraocular pressure (IOP) is the main risk factor, the exact pathology remained indistinguishable. Therefore, it is necessary to have appropriate models to investigate these mechanisms. Here, we analysed a transgenic glaucoma mouse model (βB1‐CTGF) to elucidate new possible mechanisms of the disease. Therefore, IOP was measured in βB1‐CTGF and wildtype mice at 5, 10 and 15 weeks of age. At 5 and 10 weeks, the IOP in both groups were comparable (*P* > 0.05). After 15 weeks, a significant elevated IOP was measured in βB1‐CTGF mice (*P* < 0.001). At 15 weeks, electroretinogram measurements were performed and both the a‐ and b‐wave amplitudes were significantly decreased in βB1‐CTGF retinae (both *P* < 0.01). Significantly fewer Brn‐3a^+^ retinal ganglion cells (RGCs) were observed in the βB1‐CTGF group on flatmounts (*P* = 0.02), cross‐sections (*P* < 0.001) and also via quantitative real‐time PCR (*P* = 0.02). Additionally, significantly more cleaved caspase 3^+^ RGCs were seen in the βB1‐CTGF group (*P* = 0.002). Furthermore, a decrease in recoverin^+^ cells was observable in the βB1‐CTGF animals (*P* = 0.004). Accordingly, a significant down‐regulation of *Recoverin* mRNA levels were noted (*P* < 0.001). *Gfap* expression, on the other hand, was higher in βB1‐CTGF retinae (*P* = 0.023). Additionally, more glutamine synthetase signal was noted (*P* = 0.04). Although no alterations were observed regarding photoreceptors via immunohistology, a significant decrease of *Rhodopsin* (*P* = 0.003) and *Opsin* mRNA (*P* = 0.03) was noted. We therefore assume that the βB1‐CTGF mouse could serve as an excellent model for better understanding the pathomechanisms in POAG.

## 1 INTRODUCTION

An elevated intraocular pressure (IOP) is considered the main risk factor for glaucoma.^1^ This neurodegenerative disease is defined as an optic neuropathy with changes at the optic nerve head and progressive retinal ganglion cell (RGC) death followed by visual field loss.^1^ Glaucoma usually remains asymptomatic until late‐stage disease, when about 30% of the RGCs are lost. Then, abnormalities in automated visual field testing can be noted.^2^ The current therapies aim to lower the IOP. However, these treatments can only decelerate the progression of the RGC death and not recover cells which were already damaged. Therefore, it will be beneficial to detect glaucoma at very early stages to begin therapies as soon as possible. To acquire this goal, an adequate animal model for primary open‐angle glaucoma (POAG) is required. Several ocular hypertension (OHT) models are available, but most of them elevate the IOP through surgical interventions.^3^, ^4^, ^5^, ^6^ These manipulations could lead to local inflammations and therefore would be detrimental for analyses of the immune response. As genetic model, the DBA2/J mouse is used most of the time.^7^, ^8^, ^9^ However, this model more reflects a pigment dispersion glaucoma.^7^, ^10^, ^11^ A new transgenic model for POAG is the connective tissue growth factor (βB1‐CTGF) mouse. Here, the lens‐specific overexpression of CTGF leads to changes of the extracellular matrix and of the cytoskeleton in the trabecular meshwork followed by an increase of the IOP. Furthermore, a progressive loss of axons in the βB1‐CTGF optic nerves was reported after 4‐12 weeks.^12^ In the study presented here, we aimed to investigate functional and morphological changes in the retina of the βB1‐CTGF mice in comparison to IOP changes for the first time. We therefore performed functional analyses of the retina. Retinal samples were investigated regarding different cell types including RGCs, bipolar cells, macroglia and photoreceptors. We could demonstrate that an elevated IOP is associated with the apoptotic loss of RGCs in the transgenic βB1‐CTGF mouse model.

## 2 METHODS

### 2.1 Animals

Transgenic βB1‐Crystallin‐CTGF mice were generated as described in detail previously.^12^ In brief, for generation of the βB1‐Crystallin‐CTGF construct, the murine cDNA of CTGF was cloned in a plasmid containing the βB1‐Crystallin promoter and the simian virus 40 (SV40) polyA signal region and the SV40 small‐T intron to obtain plasmid βB1‐Crystallin‐CTGF. Constructs for microinjection were released from plasmid pβB1‐CTGF by digestion and transgenic mice were generated in a FVB/N background as described previously.^13^ For this study, the mice were backcrossed into a CD1 background over eight generations. Therefore both, wildtype (WT) and βB1‐CTGF animals, had a CD1 background. All animals were bred in‐house at the animal facility at the Ruhr‐University Bochum. WT CD1 mice for breeding were obtained from Charles River. βB1‐CTGF mice for breeding were kindly provided by Prof. Fuchshofer (University Regensburg, Germany). Potential βB1‐CTGF mice were screened by isolating genomic DNA from tail biopsies and testing for transgenic sequenced by PCR, using the following primer sequences: 5'‐GGAAGTGCCAGCTCATCAGT‐3' and 5'‐GTGCGGGACAGAAACCTG‐3'. 15 weeks old female and male mice were included in the study. All procedures concerning animals adhered to the ARVO statement for the use of animals in ophthalmic and vision research. All experiments involving animals were approved by the animal care committee of North Rhine‐Westphalia, Germany. Mice were kept under environmentally controlled conditions with free access to chow and water.

### 2.2 Measurement of IOP

Intraocular pressure of both eyes and both groups was measured at 5, 10 and 15 weeks (n = 6‐10 animals/group) using a rebound tonometer (TonoLab, Icare) as described previously.^14^, ^15^ For this procedure, mice were anaesthetized with a ketamine/xylazine cocktail (120/16 mg/kg). All measurements were performed by one examiner at the same time of the day. For each analysis, the mean of 10 measurements was calculated.

### 2.3 Electroretinogram analyses

For electroretinogram (ERG) measurements, mice were dark adapted overnight. The retinal function was monitored in both eyes using full‐field‐flash electroretinography (HMsERG system, OcuScience LLC) after 15 weeks as described previously (n = 8 animals/group).^16^, ^17^ Mice were anaesthetized with a ketamine/xylazine cocktail (120/16 mg/kg) and eyes were dilated with tropicamide (5%) and topically anaesthetized using conjuncain. Body temperature was maintained at 37°C with a feedback temperature controller (TC‐1000; CWE Inc). Reference electrodes were placed subcutaneously below the right and left ear and a ground electrode was placed in the base of the tail. Contact lenses with silver thread recording electrodes were placed in the center of the cornea after application of methocel (Omni Vision). Before measurement, the electroretinography equipment was covered with a faraday cage. Scotopic flash ERGs were recorded at 0.1, 0.3, 1, 3, 10 and 25 cd.s/m^2^. For the light intensities of 0.1‐3 cd.s/m^2^, four flashes were averaged and for 10 and 25 cd.s/m^2^, one flash was measured. The interstimulus interval was 10 seconds between flashes of the same light intensity. Signals obtained from the corneal surface were amplified, digitized, averaged and stored using commercial software (ERGView 4.380R; OcuScience LLC). A 50 Hz filtering of the data was applied before evaluating the amplitude of the a‐ and b‐wave.

### 2.4 Retinal ganglion cell counts via flatmounts

At 15 weeks, eyes were fixed in 4% paraformaldehyde for 1 hour and then prepared as flatmounts (n = 12 eyes/group).^18^ Briefly, flatmounts were blocked with 10% donkey serum in 0.5% Triton‐X in PBS for 90 minutes. Afterwards, they were incubated with the RGC marker Brn‐3a (1:300, Santa Cruz) overnight. The corresponding secondary antibody, donkey anti‐goat Alexa Fluor 488 (1:1000, Dianova), was added the next day for 2 hours. From each of the four flatmount arms, three photos were captured (central, middle, peripher) with an Axio Imager M2 fluorescence microscope (Zeiss). Brn‐3a^+^ cells were counted using ImageJ software (NIH).

### 2.5 Immunohistology

In order to identify different retinal cell types, specific immunofluorescence antibodies were applied (n = 5‐9 eyes/group, 6 sections/staining; Table 1).^18^ Briefly, retina cross‐sections were blocked with a solution containing 10%–20% donkey, 2%–3% BSA and/or goat serum and 0.1% Triton‐X in PBS. For GFAP, 0.2% cold water fish gelatin was added. Primary antibodies were incubated at room temperature overnight. Incubation using corresponding secondary antibodies was performed for 1 hour on the next day. Nuclear staining with 4',6 diamidino‐2‐phenylindole (DAPI, Serva Electrophoresis) was included to facilitate the orientation on the slides. Negative controls were performed by using secondary antibodies only.

**Table 1:** Primary and secondary antibodies used for immunohistochemistry

Primary antibodies			Secondary antibodies		
Antibody	Company	Dilution	Antibody	Company	Dilution
Anti‐Brn‐3a	Santa Cruz	1:100	Donkey anti‐goat Alexa Fluor 488	Dianova	1:500
Anti‐cleaved caspase 3	Sigma‐Aldrich	1:300	Donkey anti‐rabbit Alexa Fluor 555	Invitrogen	1:500
Anti‐GFAP	Life Span Bioscience	1:2000	Goat anti‐chicken Alexa Fluor 488	Molecular probes	1:1000
Anti‐glutamine synthetase	Santa Cruz	1:250	Rabbit anti‐goat Cy3	Jackson Immuno research	1:2000
Anti‐opsin	Millipore	1:1200	Donkey anti‐rabbit Alexa Fluor 555	Invitrogen	1:500
Anti‐PKCα	Santa Cruz	1:300	Goat anti‐mouse Alexa Fluor 488	Invitrogen	1:500
Anti‐rhodopsin	Abcam	1:400	Goat anti‐mouse Alexa Fluor 488	Invitrogen	1:500
Anti‐vimentin	Sigma‐Aldrich	1:100	1. Biotinylated anti‐goat IgG	Vector laboratories	1:500
			2. Streptavidin Alexa Fluor 555	Life technologies	1:1000

### 2.6 Histological examination

All photographs were taken using a fluorescence microscope (Axio Imager M1 or M2). Two photos of the peripheral and two of the central part of each section were captured. The images were transferred to Corel Paint Shop Pro (V13, Corel Corporation) and equal excerpts were cut out.^19^ Afterwards, Brn‐3a^+^, PKCα^+^ and opsin^+^ cells were counted using ImageJ software. Regarding GFAP, glutamine synthetase, vimentin, rhodopsin and recoverin, area analyses were performed using an ImageJ macro.^19^, ^20^ Briefly, images were transformed into grayscale. To minimize interference with background labeling, a defined rolling ball radius was subtracted (Table 2). Then, for each picture, a suitable lower threshold was set. The ideal threshold was obtained when the grayscale picture and the original one corresponded (Table 2). Afterwards, the mean value of the lower threshold was calculated, and this number was used for final analysis. The percentage of the labelled area was measured between these defined thresholds.

**Table 2:** Adjustments of ImageJ macro for area analysis. The background subtraction as well as the lower and the upper thresholds are listed

Protein	Background subtraction (pixel)	Lower threshold	Upper threshold
GFAP	50	19.19	158.58
Glutamine synthetase	50	12.62	252.62
Recoverin	50	9.95	264
Rhodopsin	50	9.02	265
Vimentin	20	20.31	84.81

### 2.7 Quantitative real‐time PCR

Both retinae of each animal (five animals/group) were pooled for RNA preparation and cDNA synthesis as previously described.^18^, ^21^ The designed oligonucleotides for Quantitative real‐time PCR (qRT‐PCR) are shown in Table 3. *Β‐actin* and *Cyclophilin* served as reference genes. The qRT‐PCR was performed using DyNAmo Flash SYBR Green (Thermo Scientific) on the PikoReal qRT‐PCR Cycler (Thermo Scientific).^17^, ^22^

**Table 3:** Sequences of oligonucleotides. The listed oligonucleotide pairs were used in quantitative real‐time PCR experiments, while *Β‐actin* and *Cyclophilin* served as reference genes. The predicted amplicon sizes are given

Gene	Forward (F) and reverse (R) oligonucleotides	GenBank acc. no.	Amplicon size
*Β‐actin*‐F *Β‐actin*‐R	ctaaggccaaccgtgaaag accagaggcatacagggaca	NM_007393.5	104 bp
*Cyclophilin*‐F *Cyclophilin*‐R	ttcttcataaccacaagtcaagacc tccacctccgtaccacatc	M60456.1	95 bp
*Gfap*‐F *Gfap*‐R	acagactttctccaacctccag ccttctgacacggatttggt	NM_010277.3	63 bp
*Opsin*‐F *Opsin*‐R	ccgctatctgaagatctgttatctg tgccaggcagatgtaggc	AY318865.1	73 bp
*Pkcα*‐F *Pkcα*‐R	caagggatgaaatgtgacacc cctcttctctgtgtgatccattc	NM_011101.3	96 bp
*Pou4f1*‐F *Pou4f1*‐R	ctccctgagcacaagtaccc ctggcgaagaggttgctc	AY706205.1	98 bp
*Recoverin*‐F *Recoverin*‐R	caatgggaccatcagcaaa cctaggcttgatcattttga	NM_009038.2	71 bp
*Rhodopsin*‐F *Rhodopsin*‐R	tgtggtcttcacctggatcat gaacattgcatgccctcag	NM_145383.1	90 bp

Abbreviations: F, forward; R, reverse; acc. no, accession number; bp, base pair.

### 2.8 Statistics

Intraocular pressure, ERG, and immunhistological data are presented as mean ± SEM. The βB1‐CTGF animals were compared to the WT group via two‐tailed Student's *t* test using Statistica Software (Version 13, Dell). Regarding qRT‐PCR, the relative expression values are presented as median±quartile+minimum/maximum and were assessed via Pair Wise Fixed Reallocation Randomisation Test© using REST© software (Qiagen).^18^, ^21^, ^23^, ^24^ *P*‐values below 0.05 were considered statistically significant, *P < 0.05, **P < 0.01, ***P < 0.001.

## 3 RESULTS

### 3.1 Increase of IOP

**Figure 1:**
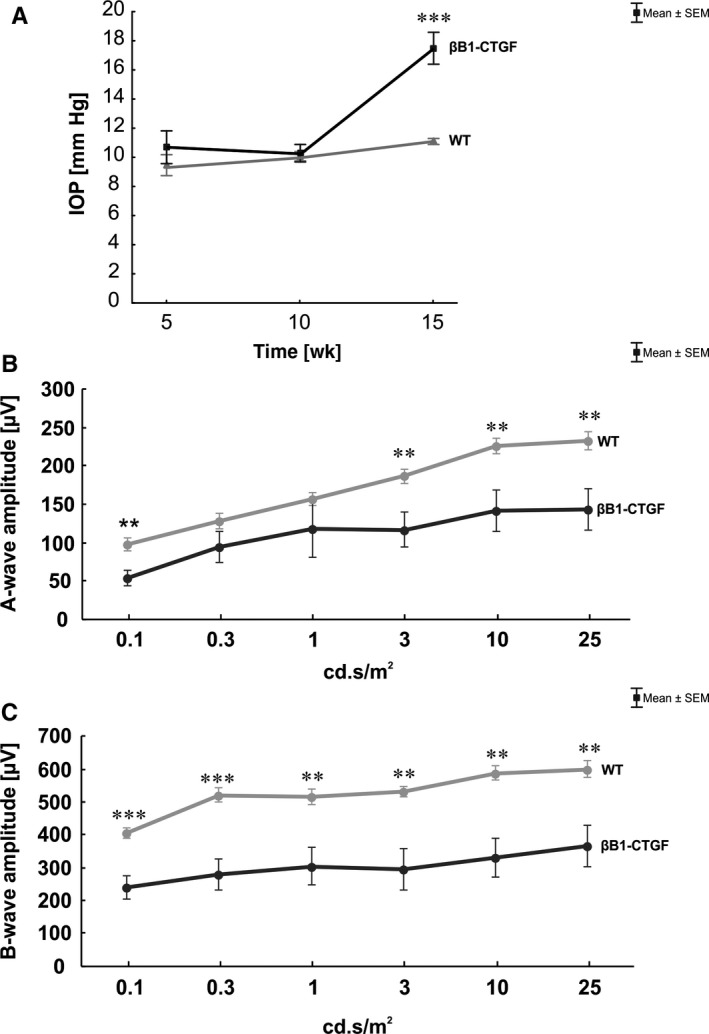
(A), At 5, 10 and 15 wk, intraocular pressure (IOP) was measured in βB1‐CTGF and wildtype (WT) animals. No changes regarding the IOP were notable in both groups after 5 and 10 wk (*P* > 0.05). At 15 wk, a significantly increased IOP was observed in the βB1‐CTGF group (*P* < 0.001). (B), After 15 wk, electroretinogram (ERG) measurements were performed. The a‐wave amplitude of the βB1‐CTGF animals was significantly decreased at a light intensity of 0.1 cd.s/m^2^ (*P* = 0.004) and from 3 to 25 cd.s/m^2^ (*P* < 0.01). (C), Also, a significantly diminished b‐wave amplitude was noted in the βB1‐CTGF group compared to WT at all light intensities (*P* < 0.01). Values are mean ± SEM. **P < 0.01, ***P < 0.001. IOP: n = 6‐10 animals/group; ERG: n = 8 animals/group

Intraocular pressure was measured after 5, 10 and 15 weeks (Figure 1A). We could not measure significant changes in the IOP between βB1‐CTGF and WT animals after 5 weeks (βB1‐CTGF: 10.69 ± 1.13 mm Hg; WT: 9.57 ± 0.49 mm Hg; *P* = 0.3) and 10 weeks (βB1‐CTGF: 10.28 ± 0.60 mm Hg; WT: 10.46 ± 0.25 mm Hg; *P* = 0.8). At 15 weeks, a significantly increased IOP could be observed in βB1‐CTGF animals (17.49 ± 1.09 mm Hg) compared to the WT littermates (11.09 ± 0.20 mm Hg; *P* < 0.001).

### 3.2 Decrease of retinal function

At 15 weeks, ERG analyses were performed and all results of the a‐ and b‐wave amplitudes are shown in Table 4. The a‐wave amplitude represents the electrical output of the photoreceptors. At 0.1 cd.s/m^2^, we noted a significant decrease of the a‐wave amplitude in βB1‐CTGF retinae compared to  control (*P* = 0.004). No changes were noted at the light intensities of 0.3 cd.s/m^2^ (*P* = 0.2) and 1 cd.s/m^2^ (*P* = 0.3). However, a significantly reduced a‐wave response was observed in βB1‐CTGF animals compared to WT (*P* = 0.01) at 3 cd.s/m^2^. Also, at a light intensity of 10 cd.s/m^2^, a decreased a‐wave amplitude was observed (*P* = 0.01). This reduction was also noted in βB1‐CTGF animals compared to WT (*P* = 0.009) at 25 cd.s/m^2^ (Figure 1B).

**Table 4:** Summary of electroretinogram results. For all light intensities, the mean a‐ and b‐wave amplitudes of wildtype (WT) and βB1‐CTGF animals and the respective p‐values are shown

Light intensity	A‐wave amplitude [µV]			B‐wave amplitude [µV]		
	WT	βB1‐CTGF	*P*‐value	WT	βB1‐CTGF	*P*‐value
0.1 cd.s/m^2^	96.98 ± 8.27	52.90 ± 9.91	**0.004**	404.94 ± 14.95	238.81 ± 34.89	**<0.001**
0.3 cd.s/m^2^	127.66 ± 10.06	94.09 ± 20.24	0.2	521.60 ± 20.87	279.91 ± 47.97	**<0.001**
1 cd.s/m^2^	157.30 ± 8.56	118.01 ± 36.92	0.3	515.39 ± 23.42	304.58 ± 58.68	**0.004**
3 cd.s/m^2^	186.71 ± 9.32	116.95 ± 22.14	**0.01**	530.76 ± 15.28	295.24 ± 63.20	**0.002**
10 cd.s/m^2^	226.40 ± 9.99	142.20 ± 27.09	**0.01**	589.04 ± 22.62	330.59 ± 60.13	**0.001**
25 cd.s/m^2^	233.45 ± 12.05	143.29 ± 27.52	**0.009**	600.70 ± 25.79	365.19 ± 62.68	**0.003**

Significant *P* values are marked in bold.

The b‐wave amplitude represents the output of the inner nuclear layers. At the light intensities of 0.1 cd.s/m^2^ and 0.3 cd.s/m^2^, a significantly reduced b‐wave amplitude was observed in βB1‐CTGF retinae compared to WT (*P* < 0.001). This decrease could also be shown at 1 cd.s/m^2^ (*P* = 0.004). At a light intensity of 3 cd.s/m^2^, a decreased b‐wave amplitude is notable in βB1‐CTGF animals compared to WT (*P* = 0.002). The b‐wave response was significantly reduced in βB1‐CTGF retinae compared to WT (*P* = 0.001) at 10 cd.s/m^2^. Also, at 25 cd.s/m^2^, the b‐wave amplitude was significantly decreased in βB1‐CTGF animals compared to WT animals (*P* = 0.003) (Figure 1C).

### 3.3 Loss of retinal ganglion cells

**Figure 2:**
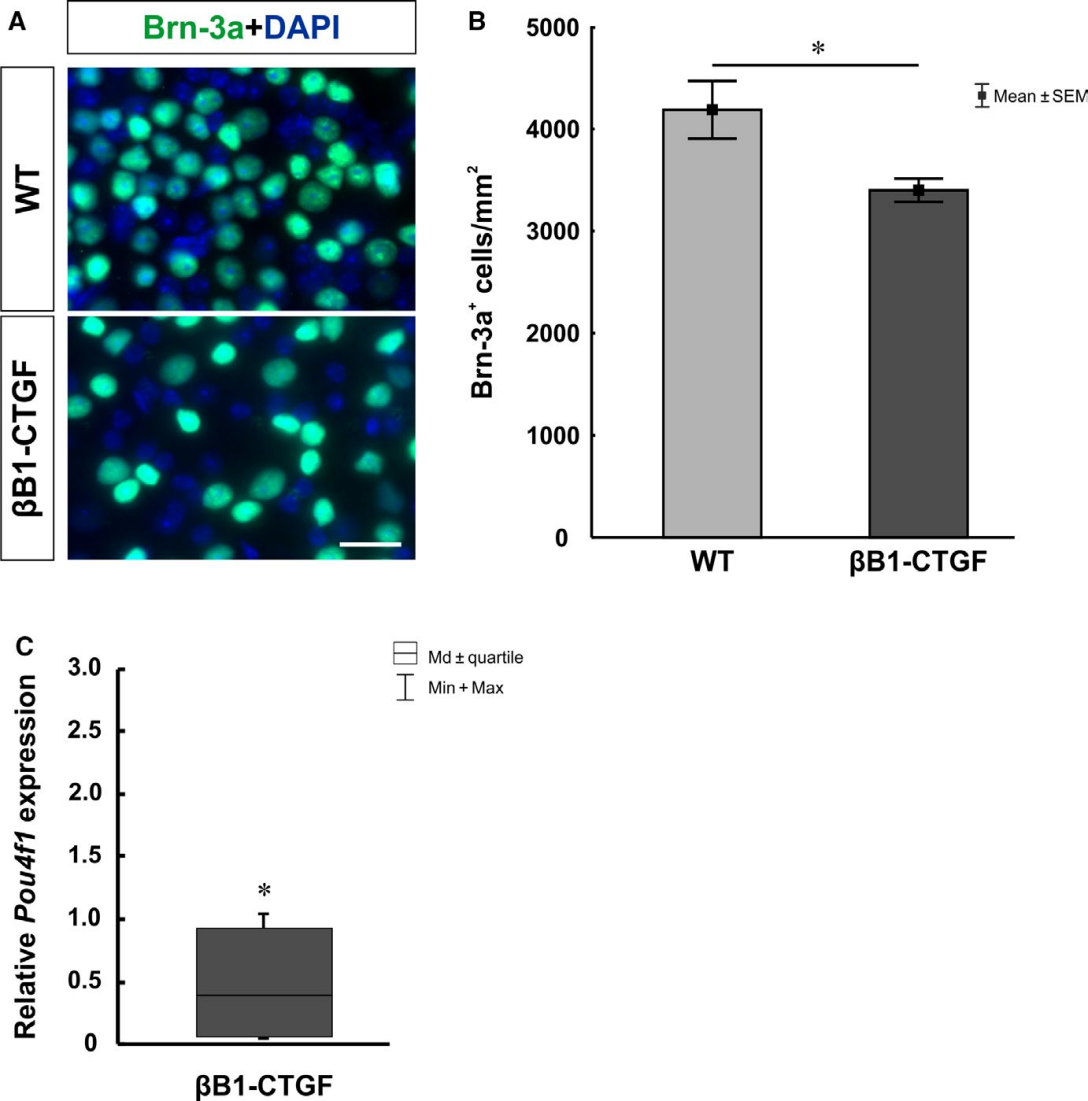
(A), Flatmounts of both groups were labelled with the retinal ganglion cell marker Brn‐3a (green) after 15 wk. (B), βB1‐CTGF retinae revealed a significant loss of Brn‐3a^+^ cells compared to wildtype (*P* = 0.02). (C), QRT‐PCR analyses showed a down‐regulation of *Pou4f1* mRNA levels in βB1‐CTGF animals (*P* = 0.02). Values are mean ± SEM for immunohistology and median±quartile+maximum/minimum for qRT‐PCR. Scale bar: 20 µm. *P < 0.05. Flatmounts: n = 12 eyes/group; qRT‐PCR: n = 5 animals/group

To evaluate a possible alteration in the number of RGCs, flatmounts were stained with an antibody against Brn‐3a^18^ (Figure 2A). Additionally, qRT‐PCR analyses were performed regarding the mRNA level of *Pou4f1* (Figure 2C). Significantly fewer Brn‐3a^+^ cells were noted in βB1‐CTGF animals (3399.39 ± 114.57 cells/mm^2^) in comparison to WT (4186.92 ± 282.94 cells/mm^2^; *P* = 0.02) after 15 weeks (Figure 2B). Furthermore, a significant down‐regulation was observed in *Pou4f1* mRNA levels at this age (0.40‐fold; *P* = 0.02).

**Figure 3:**
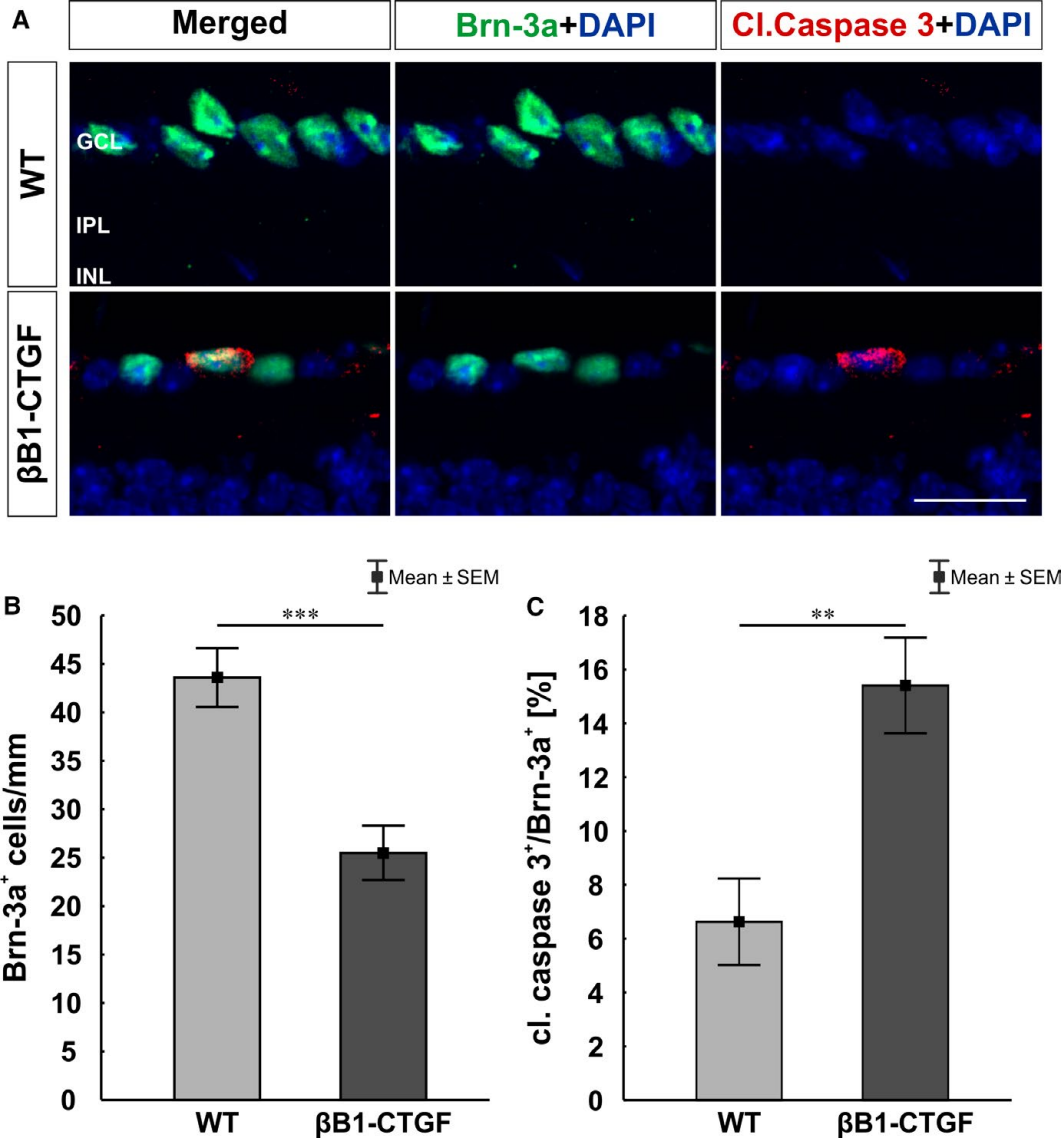
(A), Retinal cross‐sections were stained against Brn‐3a (green) and the apoptosis marker cleaved caspase 3 (red) at 15 wk. Cell nuclei were visualized with DAPI (blue). (B), The number of Brn‐3a^+^ cells was significantly decreased in the βB1‐CTGF mice compared to wildtype (WT) mice (*P* < 0.001). (C), Additionally, significantly more cleaved caspase 3^+^ retinal ganglion cells were revealed in βB1‐CTGF mice compared to WT animals (*P* = 0.002). GCL, ganglion cell layer; INL, inner nuclear layer; IPL, inner plexiform layer. Values are mean ± SEM. Scale bar: 20 μm. **P < 0.01, ***P < 0.001. N = 9 eyes/group

To confirm the loss of RGCs, cross‐sections were labeled with anti‐Brn‐3a. To detect a possible apoptosis of RGCs, co‐staining with an antibody against cleaved caspase 3 was performed (Figure 3A). We noted a loss of RGCs in βB1‐CTGF retinae (25.50 ± 2.80 cells/mm) compared to WT animals (43.59 ± 3.03 cells/mm; *P* = 0.0005; Figure 3B). Additionally, significantly more cleaved caspase 3^+^ RGCs were observed in the βB1‐CTGF group (15.41 ± 1.78%) in comparison to WT retinae (6.63 ± 1.60%; *P* = 0.002; Figure 3C).

### 3.4 Macroglia activation

**Figure 4:**
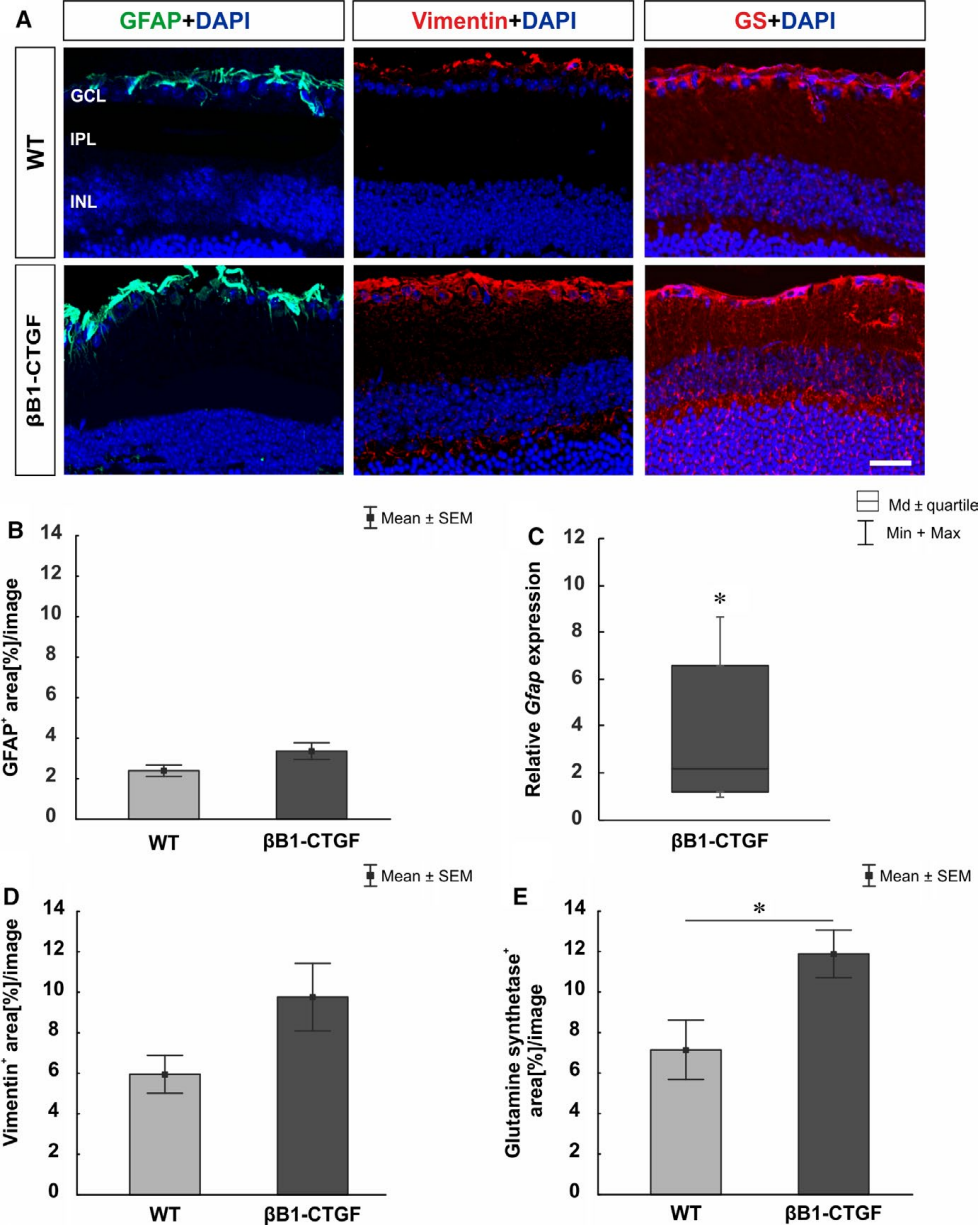
(A), Macroglia were stained using GFAP (green), vimentin (red) and glutamine synthetase (red) labelling. DAPI (blue) visualized cell nuclei. (B), A slight trend towards an enhanced GFAP signal was noted in βB1‐CTGF animals (*P* = 0.09). (C), However, a significant up‐regulation of *Gfap* mRNA levels were noted in βB1‐CTGF retinae in comparison to wildtype (WT) (*P* = 0.02). (D), In βB1‐CTGF retinae, a trend towards more vimentin^+^ signal was noted (*P* = 0.08). (E), Regarding glutamine synthetase, we observed significantly larger staining area in βB1‐CTGF mice compared to WT (*P* = 0.04). GCL, ganglion cell layer; GS, glutamine synthetase; INL, inner nuclear layer; IPL, inner plexiform layer. Values are mean ± SEM for immunohistology and median±quartile+maximum/minimum for qRT‐PCR. Scale bar: 20 μm. *P < 0.05. Immunohistology: n = 5 eyes/group; qRT‐PCR: n = 5 animals/group

A possible macrogliosis was investigated by labeling retinae against anti‐GFAP, anti‐vimentin and anti‐glutamine synthetase (Müller glia and astrocytes; Figure 4A). Furthermore, the mRNA expression levels of *Gfap* was evaluated via qRT‐PCR (Figure 4C). The GFAP area analysis showed a slight trend to more GFAP signal in βB1‐CTGF mice (3.36 ± 0.41 area [%]/section) compared to WT (2.39 ± 0.28 area [%]/section; *P* = 0.09; Figure 4B). However, qRT‐PCR analyses revealed a significant up‐regulation of *Gfap* expression levels in the βB1‐CTGF retinae compared to WT (2.2‐fold; *P* = 0.02; Figure 4C). Furthermore, a trend towards more vimentin^+^ area was observed in βB1‐CTGF retinae (WT: 5.95 ± 0.93 area [%]/section; βB1‐CTGF: 9.76 ± 1.67 area [%]/section; *P* = 0.08; Figure 4D). Significantly more glutamine synthetase signal was noted in βB1‐CTGF animals (11.89 ± 1.17 area [%]/section) in comparison to WT (7.15 ± 1.47 area [%]/section; *P* = 0.04; Figure 4E).

### 3.5 Decrease in cone bipolar cells

**Figure 5:**
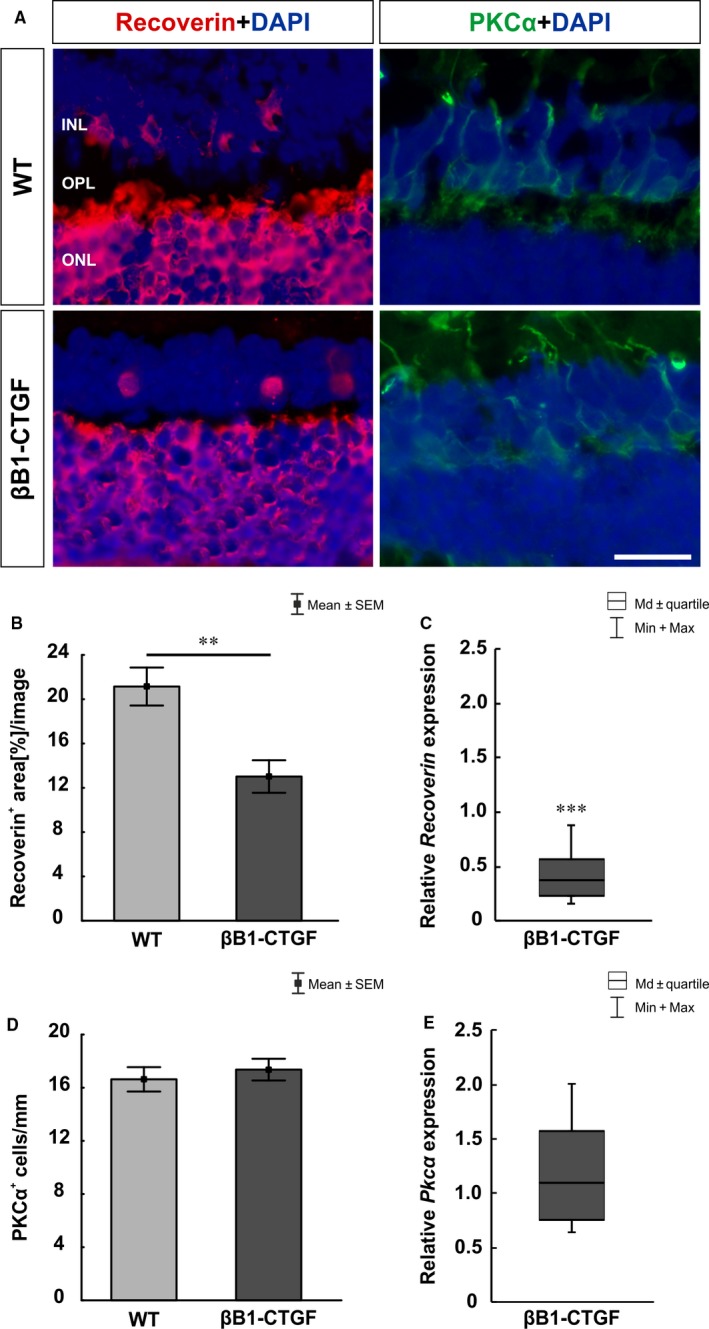
(A), Cone bipolar cells were evaluated by using recoverin (red) and rod bipolar cells using PKCα staining (green). Cell nuclei were labelled with DAPI (blue). (B), The analyses of the recoverin^+^ area revealed a significantly decreased staining area in βB1‐CTGF animals (*P* = 0.004). (C), The analyses of *Recoverin* mRNA expression showed a significant down‐regulation in the βB1‐CTGF group (*P* < 0.001). (D), PKCα^+^ cells were not altered in βB1‐CTGF retinae compared to wildtype (WT) (*P* > 0.05). (E), The mRNA expression levels of *Pkcα* revealed no changes in βB1‐CTGF retinae (*P* = 0.6). INL, inner nuclear layer; ONL, outer nuclear layer; OPL, outer plexiform layer. Values are mean ± SEM for immunohistology and median±quartile+maximum/minimum for qRT‐PCR. Scale bar: 20 μm. **P < 0.01, ***P < 0.001. Immunohistology: n = 7 eyes/group; qRT‐PCR: n = 5 animals/group

To evaluate the number of bipolar cells, retinae were labelled with anti‐PKCα (rod bipolar cells) and anti‐recoverin (cone bipolar cells; Figure 5A). Additionally, qRT‐PCR analyses were performed regarding the mRNA levels of *Pkcα* and *Recoverin* (Figure 5C,E). We noted a significantly smaller recoverin^+^ signal area in retinae of the βB1‐CTGF group (13.02 ± 1.47 area [%]/section) in comparison with WT mice (21.15 ± 1.74 area [%]/section; *P* = 0.004; Figure 5B). Furthermore, the qRT‐PCR analyses showed a significant down‐regulation of *Recoverin* mRNA levels in βB1‐CTGF animals (0.37‐fold expression; *P* < 0.001; Figure 5C). Staining with PKCα revealed no changes in the βB1‐CTGF group (17.36 ± 0.82 cells/mm) compared to WT (16.63 ± 0.92 cells/mm; *P* > 0.05; Figure 5D). Also, we noted no changes in *Pkcα* mRNA levels in βB1‐CTGF retinae (1.1‐fold expression; *P* = 0.6; Figure 5E).

### 3.6 Effects on photoreceptors

**Figure 6:**
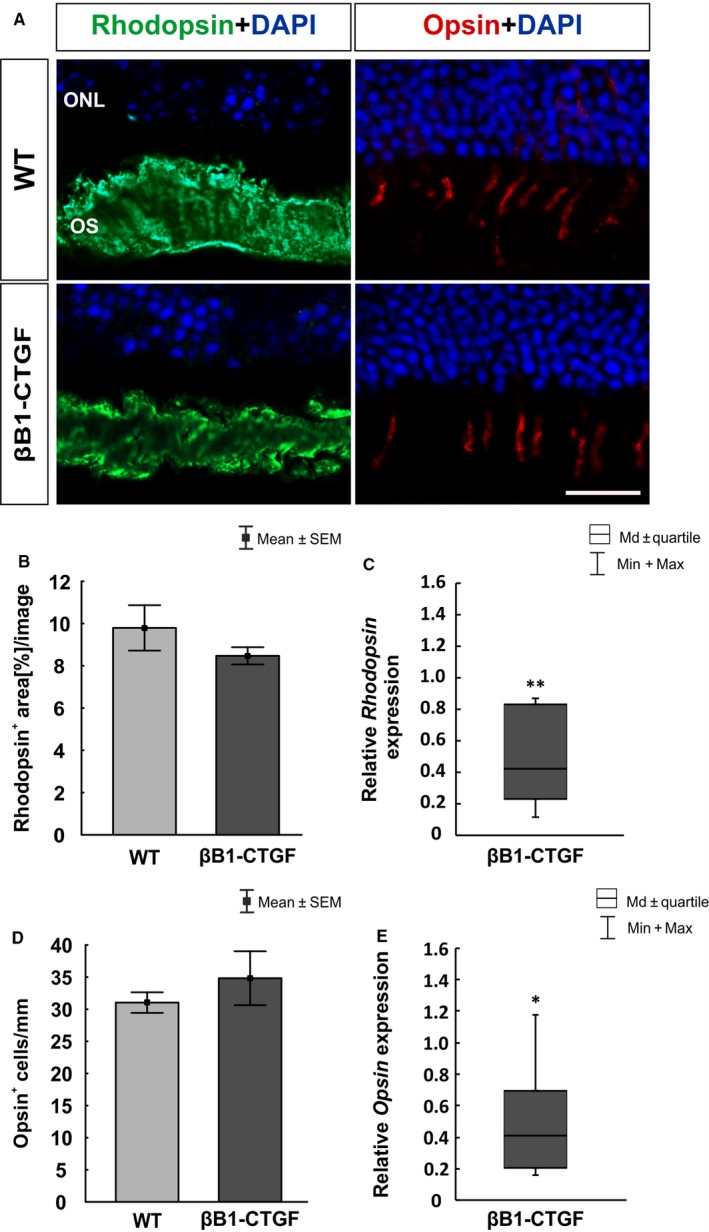
(A), Rhodopsin (rods, green) and opsin (L‐cones, red) staining of retinal sections was performed to evaluate photoreceptors. DAPI was added to visualize cell nuclei (blue). (B), Comparable rhodopsin^+^ areas were detected in both groups (*P* > 0.05). (C), However, qRT‐PCR analyses revealed a significant down‐regulation of *Rhodopsin* mRNA expression levels in βB1‐CTGF retinae (*P* = 0.003). (D), The number of opsin^+^ cells remained unaltered in βB1‐CTGF animals compared to wildtype (WT) (*P* > 0.05). (E), The mRNA expression levels of *Opsin* were significantly down‐regulated in in βB1‐CTGF retinae (*P* = 0.03). ONL, outer plexiform layer; OS, outer segment. Values are mean ± SEM for immunohistology and median±quartile+maximum/minimum for qRT‐PCR. Scale bar: 20 μm. *P < 0.05, **P < 0.01. Immunohistology: n = 7 eyes/group; qRT‐PCR: n = 5 animals/group

At 15 weeks, possible alterations regarding the photoreceptors was investigated. Therefore, L‐cones were labelled with anti‐opsin and rods were visualized with anti‐rhodopsin (Figure 6A). Furthermore, *Rhodopsin* and *Opsin* mRNA expression levels were analysed via qRT‐PCR (Figure 6C,E). The density of the rhodopsin^+^ area was comparable in both groups (WT: 9.43 ± 0.88 area [%]/section; βB1‐CTGF: 8.51 ± 0.44 area [%]/section; *P* > 0.05; Figure 6B). However, a significant down‐regulation of *Rhodopsin* mRNA levels was noted in βB1‐CTGF animals (0.42‐fold expression; *P* = 0.003; Figure 6C). No changes were observed in the number of opsin^+^ cells in βB1‐CTGF retinae (33.38 ± 3.42 cells/mm, *P* > 0.05) compared to WT (31.28 ± 1.51 cells/mm; Figure 6D). The qRT‐PCR analyses revealed a significant down‐regulation of *Opsin* mRNA levels in βB1‐CTGF animals (0.41‐fold expression; *P* = 0.03; Figure 6E).

## 4 DISCUSSION

The pathomechanisms leading to glaucoma are still not known precisely. However, an elevated IOP has been consistently associated with the prevalence^25^, ^26^, ^27^, ^28^ and incidence^29^, ^30^, ^31^ of open‐angle glaucoma.^1^ In most western countries, approximately half of the patients with manifest glaucoma are not diagnosed.^1^, ^32^, ^33^ To improve both the treatment and the diagnosis of glaucoma patients, it is necessary to have appropriate models. The βB1‐CTGF mouse seems to be a promising tool to analyze mechanisms in POAG. In 2012, Junglas et al reported that the overexpression of CTGF led to an increased IOP accompanied with a progressive loss of optic nerve axons.^12^ In this study, the transgenic βB1‐CTGF mice also developed an elevated IOP after 15 weeks. This is diverging to previous results, where an increased IOP was noted already after 1 month.^12^ These varieties could be explained by different environments and mouse strains. In the original paper, the mice were analysed in a mixed background of FVBN/CD1 in the first generation, whereas the mice used in this study were in a pure CD1 background. The CD1‐strain is an out‐bred strain with a higher genetic variability. Furthermore, it is described that the same experiment, performed in two laboratories, can lead to a discrepancy in the results.^34^, ^35^ Nevertheless, we could constantly measure an increase of the IOP in βB1‐CTGF mice. In this study, we focused on IOP‐dependent damage in the retina. Simultaneously to the observed IOP elevation, we could also detect an apoptotic loss of RGCs at 15 weeks. Based on the results of the initial study, we conclude that the IOP dependent axon loss in the optic nerve leads than to a consecutive loss of RGCs. The time course of progression in the βB1‐CTGF mice would be similar to the development of glaucoma disease in patients.^1^, ^36^

The majority of retinal diseases are associated with reactive gliosis.^37^, ^38^, ^39^ The astrocytes and Müller glia cells become reactive during the pathogenesis of glaucoma, characterized by morphologic alterations and expression changes.^40^, ^41^, ^42^ We found that the observed loss of RGC cells is accompanied by changes of the macroglia cells in the retinae of the βB1‐CTGF mice. The immunohistological analysis showed a slight increase of astrocytic markers like GFAP and vimentin, but we found a profound increase of *GFAP* mRNA in the βB1‐CTGF mice. We assume that the changes could be the start of a remodelling process in the retinal astrocytes based on the IOP and the RGC loss. It is known that astrocytes of the optic nerve head respond strongly to glaucomatous damage.^43^ In retinal diseases, the induction of GFAP in Müller cells is an early and very sensitive marker for reactive Müller cells, which is often accompanied by an increased expression of glutamine synthetase.^44^ Accordingly, in the eyes of human donors suffering from glaucoma, an increased expression of GFAP in Müller cells has been detected.^45^ In our study, the Müller glial cells showed a GFAP positive signal in the βB1‐CTGF mice in comparison to the WT littermates. As the Müller glial signal of the GFAP staining had an overlap with the signal of the astrocytes it is rather difficult to quantify the differences. To overcome this problem, we stained against glutamine synthetase in both groups. The Müller glial cells had a significant enhanced signal for glutamine synthetase, which emphasize a reactive state of the Müller glial cells. The enhanced reactivity of Müller glial cells could be a response to the IOP induced RGC loss and can mediate both protective as well as detrimental effects on retinal neurons.^44^

To characterize the βB1‐CTGF model more precisely, we additionally investigated whether an increased IOP could affect also other retinal layers. In previous studies, the induction of OHT in mice of the albino Swiss strain caused a reduced recoverin immunoreactivity and the number of PKCα^+^ rod bipolar cells was diminished.^46^ Similar results were observed in a rat model after IOP elevation, were a decrease of rod bipolar cells was reported 5 weeks after IOP elevation.^47^ In our study, we could only observe a diminished cone bipolar cell signal at 15 weeks of age in the retinae of the βB1‐CTGF in comparison to their WT littermates. The different results regarding the rod bipolar cells could be explained by the different experimentally and genetically mechanisms causing the increased IOP. In the βB1‐CTGF mouse, cone bipolar cells seem to be more sensitive to IOP changes. Nevertheless, ERG data from our study suggest a functional loss of inner nuclear cells and photoreceptors in βB1‐CTGF animals. In glaucoma patients, ERG measurements revealed a degeneration of photoreceptor cells in late stages of glaucoma.^48^, ^49^ Also after laser coagulation in rats, a secondary loss of cones could be observed.^50^ In aged DBA2/J mice, the a‐wave amplitude was decreased, pointing towards an impairment of photoreceptors.^51^

In summary, elevated IOP is accompanied by apoptotic RGC loss and reactive gliosis in the βB1‐CTGF mice. We therefore conclude that the βB1‐CTGF mouse model can be used to study pathomechanisms occurring in POAG.

## CONFLICT OF INTEREST

The authors confirm that there are no conflict of interest.

## AUTHOR CONTRIBUTIONS

SR, DK, FF, MW and CV performed the research; SR, DK, and MW analysed the data; RF and SCJ designed the research study; SR wrote the paper; HBD, RF, and SCJ reviewed the manuscript.
